# Clinical Identification of Geriatric Patients with Hypovitaminosis D: The ‘Vitamin D Status Predictor for Geriatrics’ Study

**DOI:** 10.3390/nu9070658

**Published:** 2017-06-27

**Authors:** Cédric Annweiler, Jérémie Riou, Axel Alessandri, David Gicquel, Samir Henni, Catherine Féart, Anastasiia Kabeshova

**Affiliations:** 1 Department of Neurosciences and Aging, Division of Geriatric Medicine, Angers University Hospital, Angers University Memory Clinic, Research Center on Autonomy and Longevity, University of Angers, UPRES EA 4638, UNAM, 49035 Angers, France; anastasiia.kabeshova@gmail.com; 2 Department of Medical Biophysics, Robarts Research Institute, Schulich School of Medicine and Dentistry, the University of Western Ontario, London, ON N6A 3K7, Canada; 3 INSERM, MINT, 1066, University of Angers, 49035 Angers, France; jeremie.riou@univ-angers.fr; 4 Delegation to Clinical Research and Innovation, Angers University Hospital, 49100 Angers, France; 5 Health Faculty, School of Medicine, F-49045 Angers, France; alessandri.axel@gmail.com (A.A.); dav.gicquel@gmail.com (D.G.); 6 Department of Sports Medicine and Vascular Investigations, Angers University Hospital, 49100 Angers, France; Samir.Henni@chu-angers.fr; 7 Université de Bordeaux, ISPED, Centre INSERM U1219-Bordeaux Population Health, 61292 Bordeaux, France; catherine.feart-couret@u-bordeaux.fr

**Keywords:** screening, vitamin D, vitamin D deficiency, older adults, hospital related

## Abstract

The 16-item Vitamin D Status Predictor (VDSP) tool identifies healthy older community-dwellers at risk of hypovitaminosis D and may guide the use of blood tests in this population. The objective of the present hospital-based study was to test the efficacy of the VDSP to identify geriatric patients with hypovitaminosis D. The study included 199 nonsupplemented geriatric in- and outpatients consecutively admitted to Angers University Hospital, France (mean ± SD, 82.0 ± 7.8 years; 53.3% female). Serum 25-hydroxyvitaminD (25(OH)D) was measured at the time of the physician-administered VDSP. Hypovitaminosis D was defined as serum 25(OH)D concentration ≤ 75 nmol/L for vitamin D insufficiency, 25(OH)D ≤ 50 nmol/L for vitamin D deficiency, and 25(OH)D ≤ 25 nmol/L for severe vitamin D deficiency. We found that 184 participants (92.4%) had vitamin D insufficiency, 136 (68.3%) had vitamin D deficiency, and 67 (33.7%) had severe vitamin D deficiency. The VDSP identified severe vitamin D deficiency with an area under curve (AUC) = 0.83 and OR = 24.0. The VDSP was able to identify vitamin D deficiency and vitamin D insufficiency with less accuracy (AUC = 0.71 and AUC = 0.73, respectively). In conclusion, the 16-item VDSP is a short questionnaire that accurately identifies geriatric patients with severe vitamin D deficiency. This tool may guide the use of blood collection for determining geriatric patients’ vitamin D status.

## 1. Introduction

Hypovitaminosis D is common among seniors and results in multiple adverse health events [1,2]. Universal supplementation remains, however, not recommended due to potential adverse effects [3,4] and lack of evidence for cost-effectiveness [5]. A blood test is required before supplementation to confirm the presence and severity of hypovitaminosis D [6]. For this reason, the use of blood tests for the determination of serum 25-hydroxyvitamin D (25(OH)D) concentration increases dramatically, especially in older adults [7]. To prevent insurmountable health costs, new strategies should be devised to guide the use of blood tests. For instance, we recently developed among older community-dwellers the Vitamin D Status Predictor (VDSP), a 16-item questionnaire coupled with a combinatorial non-linear algorithm able to accurately identify those with hypovitaminosis D who should be administered vitamin D supplements [8]. However, this studied sample was restricted to community-dwelling older adults who were probably healthier and showed greater interest in health issues than the general population of all seniors. Based on this previous finding that it was possible to identify hypovitaminosis D among older community-dwellers without resorting to a blood test, we hypothesized that the same tool could be suitable in a hospital setting to identify hypovitaminosis D among geriatric patients, in whom hypovitaminosis D is particularly frequent and severe [1,9]. The objective of this study was to test the diagnostic efficacy of the VDSP tool for the identification of geriatric in- and outpatients with hypovitaminosis D.

## 2. Materials and Methods

### 2.1. Participants

We studied in- and outpatients aged 65 and over that were consecutively recruited in the VDSP-G (Vitamin D Status Predictor for Geriatrics) study. The VDSP-G study is an observational cross-sectional study designed to test the VDSP among all patients consecutively hospitalized or seen in consultation in the geriatric acute care unit of the University Hospital of Angers, France, from March to May 2015. After giving their informed consent for research, included participants received a full medical examination consisting of a blood test, structured questionnaires, and a standardized clinical examination. The exclusion criterion for the present analysis was the regular use of vitamin D supplements. The study was conducted in accordance with the ethical standards set forth in the Helsinki Declaration (1983). The entire study protocol was approved by the local Ethical Committee (No. 2015-03).

### 2.2. Vitamin D Status Predictor

The development of the VDSP tool was described in detail previously [8]. In summary, the VDSP is based on a non-linear model of feed forward artificial neural network (multilayer perceptron), which was built among community-dwelling older adults. All available variables in the database were introduced into the model without any *a priori* hypothesis on their possible relationship to the vitamin D status as it was their combination, but not their direct link to vitamin D, that was tested. The unnecessary variables were then deleted one by one according to their relative importance in the algorithm until the effective minimum number of variables was obtained. The final model is based on 16 clinical items [8].

Here, participants underwent a full clinical examination by a physician to collect—in a standardized manner—the following 16 items of the VDSP: gender, age (in years), number of therapeutic classes used per day, body mass index (BMI, in kg/m^2^), use walking aids, use psychoactive drugs (i.e., benzodiazepines, anti-depressants or neuroleptics), wearing glasses, sad mood, fear of falling, history of falls in the preceding year, cognitive disorders, undernutrition, polymorbidity, history of vertebral fractures, living alone, use anti-osteoporotic drugs (i.e., bisphosphonates, strontium, or calcium). The BMI was calculated based on anthropometric measurements. Undernutrition was defined as BMI below 21 kg/m^2^ [10]. Polymorbidity was defined as having more than three chronic diseases (i.e., diseases of indefinite duration or running a course with minimal change). A fall was defined as an event resulting in a person coming to rest unintentionally on the ground or at other lower level, not as the result of a major intrinsic event or an overwhelming hazard, according to the French Society of Geriatrics and Gerontology (SFGG) and French National Authority for Health [11]. The history of vertebral fractures was sought for patients’ and relatives’ interview and from the medical records. The fear of falling was sought using the following standardized question “Are you afraid of falling?”, as previously published [12]. The presence of cognitive disorders was noted based on clinical expertise and/or history of dementia from medical records. Finally, sad mood was sought using the following question from the four-item Geriatric Depression Scale: “Do you feel discouraged and sad?” [13]. Finally, and without knowledge of the blood test, we applied the algorithm previously published [8] to the items of the VDSP in order to identify among patients those with hypovitaminosis D, by distinguishing the 25, 50, and 75 nmol/L thresholds [1].

### 2.3. Serum 25(OH)D Measure

Venous blood was collected from resting participants at the time of the VDSP assessment for the measure of serum 25(OH)D concentration. All serum 25(OH)D measures were performed by radioimmunoassay (DiaSorin Inc., Stillwater, MN, USA) in a single laboratory at the University Hospital of Angers, France, according to DEQAS scheme. With this method, there is no interference of lipids, which is often observed in other non-chromatographic assays of 25(OH)D. The intra- and interassay precisions for 25(OH)D were 5.2% and 11.3%, respectively (range in normal adults aged 20–60 years, 75–312 nmol/L). Based on previous literature, three different threshold values were used consecutively to define hypovitaminosis D: vitamin D insufficiency was defined as serum 25(OH)D concentration ≤ 75 nmol/L [14], vitamin D deficiency as 25(OH)D ≤ 50 nmol/L [15], and severe vitamin D deficiency as 25(OH)D ≤ 25 nmol/L [1] (to convert to ng/mL, divide by 2.496).

### 2.4. Statistical Analysis

The participants’ characteristics were summarized using frequencies and percentages or means ± standard deviations, as appropriate. As the number of observations was higher than 40, comparisons were not affected by the shape of the error distribution and no transform was applied. Firstly, comparisons between participants separated into two groups based on serum 25(OH)D concentration (i.e., either ≤ 25 nmol/L versus > 25 nmol/L, or ≤ 50 nmol/L versus > 50 nmol/L, or ≤ 75 nmol/L versus > 75 nmol/L) were performed using Student’s *t*-test or Chi-square test, as appropriate. Secondly, univariate logistic regressions were used to examine the associations between participants’ clinical characteristics (independent variables: every single item from the VDSP tool) and hypovitaminosis D (dependent variable). Separate models were performed for each definition of hypovitaminosis D. Thirdly, the metrological properties of the complete VDSP tool (i.e., combination algorithm) were evaluated for the identification of hypovitaminosis D in this cohort of geriatric patients. *p*-values < 0.05 were considered significant. All statistics were performed using SPSS (v19.0, IBM Corporation, Chicago, IL, USA), R 3.1.0 (GNU project), NetBeans IDE 8.0, and Dag-stat [16].

## 3. Results

Among 361 eligible participants, 326 participants (90.3%) had full data available, and 199 (61.0%) participants used no vitamin D supplements and met the selection criteria (mean ± standard deviation, 82.0 ± 7.8 years; 53.3% female; 58.8% inpatient; 98% Caucasian) and were finally included in the present analysis.

The mean 25(OH)D concentration was 40 ± 23 nmol/L, and 184 participants (92.4%) had vitamin D insufficiency, 136 (68.3%) had vitamin D deficiency, and 67 (33.7%) had severe vitamin D deficiency. As illustrated in Table 1, significant differences were found between groups, especially regarding the gender, age, numbers of drugs taken daily, use of psychoactive drugs, use of anti-osteoporotic drugs, and cognitive disorders (Table 1).

Table 2 shows that only a few clinical variables were associated to hypovitaminosis D while considered separately.

**Figure 1:**
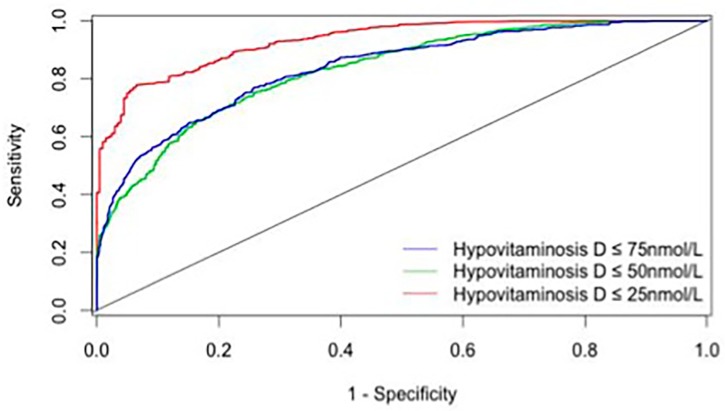
ROC curves for the identification of hypovitaminosis D with the VDSP tool, according to the different definitions of hypovitaminosis D.

Finally, Table 3 reports the metrological properties of the VDSP combinatorial algorithm for the identification of hypovitaminosis D on the whole sample. The best performance was found for the identification of severe vitamin D deficiency, with area under curve (AUC) of 0.83 on the ROC (receiver operating characteristic) curve (Figure 1). The OR for severe vitamin D deficiency was 24.0 (95% confidence interval: 9.7–59.6) while using ‘not combining variables’ as a reference. The VDSP was also able to identify vitamin D deficiency and vitamin D insufficiency with moderate efficiency (respectively, AUC = 0.71 and AUC = 0.73).

## 4. Discussion

Our results showed that the 16-item VDSP combinatorial algorithm was able to identify geriatric patients with severe vitamin D deficiency ≤ 25 nmol/L. The identification of vitamin D deficiency ≤ 50 nmol/L and vitamin D insufficiency ≤ 75 nmol/L was also possible with the VDSP, but less efficient. These results differ from those found among community-dwellers in whom the VDSP was effective in identifying mainly vitamin D insufficiency [8]. This difference can be explained by obvious health differences between general population and patients [17], and justifies distinguishing between these two populations while assessing the risk of hypovitaminosis D.

Although numerous studies have examined the variables influencing serum 25(OH)D concentration, only few have attempted to identify hypovitaminosis D among older adults, and only two among geriatric patients [18,19]. Specifically, these two previous studies have tested the performance of an isolated question [18] or a general physical questionnaire [19] to detect hypovitaminosis D among in- and outpatients; but none has evaluated the different definitions of hypovitaminosis D, and none have used a complex algorithm based on non-linear models of feed forward artificial neural networks such as the VDSP [8]. Thus, the results of the present study provide new insights into the identification of hypovitaminosis D in geriatric patients by using a novel clinical tool, and by exhibiting greater diagnostic efficacy than that hitherto demonstrated. Specifically, we found that, although each separate clinical variable exhibited only modest or no association with hypovitaminosis D (Table 2), the combination of the 16 items using the VDSP algorithm effectively identified severe vitamin D deficiency among geriatric patients. The sensitivity was particularly high (Table 3), which could make the VDSP an interesting screening tool for hypovitaminosis D, useful in guiding the use of blood collection. In this perspective, online software incorporating the VDSP algorithm is under development. The clinicians will be able to inform the 16 items of the VDSP, and the software will instantly communicate the probability of hypovitaminosis D and the opportunity to use a blood test and/or vitamin D supplements.

The identification of older adults with severe vitamin D deficiency is particularly important among geriatric patients because, since hypovitaminosis D occurs gradually, serum 25(OH)D concentrations below 25 nmol/L indicate severe and chronic hypovitaminosis D [1], which is accompanied by more severe chronic diseases [20], prolonged hospital stays [21], and higher risk of intra-hospital death [9]. For this reason, vitamin D regimens depend on the depth of the deficiency and the initial dose of supplementation should be higher in geriatric patients with severe vitamin D deficiency [6]. Thus the VDSP proves useful to identify them easily and to help supplementing them with appropriate dose.

Besides the originality of the research question on an important issue met in clinical routine, the strengths of our study include the standardized collection of data from a single research center, the testing of geriatric patients of both genders, the use of the different definitions of hypovitaminosis D used in clinical practice, and the use of a complex non-linear model of artificial intelligence. Inspired by the human brain, artificial neural networks are usually presented as interconnected systems organized in several layers, which are made up of a number of interconnected nodes containing activation function. These computational models, capable of machine learning and pattern recognition, have been developed to overcome limitations of the classical linear models [8]. Because artificial neural networks apply non-linear statistics to pattern recognition, they prove particularly adapted to multifactorial mechanisms of hypovitaminosis D. This explains why, although only a few isolated variables were associated to vitamin D status in the present study (Table 2), their combination using the VDSP combinatorial algorithm was able to identify hypovitaminosis D (Table 3). Of note, removing variables from the 16-item VDSP caused dramatic loss of the diagnostic efficiency for severe vitamin D deficiency (data not shown). Since artificial neural networks are able to learn and improve as they are fed with additional data, it is yet plausible that future versions of the VDSP may reduce the number of items required.

A number of limitations should also be acknowledged. First, the study cohort was restricted to in- and outpatients admitted to a geriatric ward who were probably frailer and with lower 25(OH)D concentrations than the population of all older patients. Second, our sample size was relatively small and could not be calculated *a priori*. Third, all tests were carried out over a relatively short period of time (March to May), which prevented examining any seasonality effect. Fourth, our findings should take into account the limitation of the 25(OH)D radioimmunoassay. Indeed, the gold standard is the liquid chromatography-tandem mass spectrometry (LC-MS/MS) [22]. However, the radioimmunoassay also offers reasonable cost, correct intra- and inter-rater reliability, and measures of 25(OH)D2 and 25(OH)D3 at once.

In conclusion, the 16-item VDSP was able to identify nonsupplemented geriatric patients with hypovitaminosis D, specifically those with severe vitamin D deficiency ≤25 nmol/L. Such an inexpensive screening tool may guide the use of blood tests to determine older adults’ vitamin D status and help clinicians in decisions to supplement their geriatric patients. In the future, the efficacy of the VDSP after the initiation of vitamin D supplements should also be questioned.

## Figures and Tables

**Table 1:** Comparison of patients’ characteristics according to vitamin D status (*n* = 199).

Clinical Characteristics	Cohort									
	Whole Sample (*n* = 199)	Serum 25(OH)D Concentration, nmol/L								
		Severe Vitamin D Deficiency ≤ 25 nmol/L (*n* = 67)	>25 nmol/L (*n* = 132)	*p*-Value *	Vitamin D Deficiency ≤ 50 nmol/L (*n* = 136)	>50 nmol/L (*n* = 63)	*p*-Value *	Vitamin D Insufficiency ≤ 75 nmol/L (*n* = 184)	>75 nmol/L (*n* = 15)	*p*-Value *
Item 1- Female gender	106 (53.3)	34 (51)	72 (55)	0.61	66 (49)	40 (64)	0.05	94 (51.1)	12 (80)	0.03 †
Item 2- Age, years (mean ± SD)	82.0 ± 7.8	83.9 ± 7.2	81.1 ± 7.9	0.01 †	82.3 ± 8.0	81.4 ± 7.4	0.41	82.1 ± 7.9	80.7 ± 7.0	0.73
Item 3- Number of drugs daily taken (mean ± SD)	5.3 ± 3.6	6.2 ± 3.8	4.8 ± 3.4	0.006 †	5.5 ± 3.7	4.8 ± 3.4	0.23	5.2 ± 3.7	5.3 ± 2.4	0.62
Item 4- Body mass index, kg/m^2^ (mean ± SD)	25.9 ± 4.5	25.3 ± 4.3	26.2 ± 4.7	0.18	26.0 ± 4.6	25.7 ± 4.4	0.65	25.8 ± 4.3	26.7 ± 7.3	0.41
Item 5- Use walking aids	89 (44.7)	34 (51)	55 (42)	0.22	62 (46)	27 (43)	0.72	82 (44.6)	7 (47)	0.88
Item 6- Use psychoactive drugs	80 (40.2)	27 (40)	53 (40)	0.98	50 (37)	30 (48)	0.15	70 (38.0)	10 (67)	0.03 †
Item 7- Wearing glasses	108 (54.3)	32 (48)	76 (58)	0.19	67 (49)	41 (65)	0.05	99 (53.8)	9 (60)	0.64
Item 8- Sad mood	62 (31.2)	22 (33)	40 (30)	0.72	39 (29)	23 (37)	0.27	55 (29.9)	7 (47)	0.18
Item 9- Fear of falling	88 (44.2)	32 (48)	56 (42)	0.47	61 (45)	27 (43)	0.79	80 (43.5)	8 (53)	0.46
Item 10- History of falls	93 (46.7)	36 (54)	57 (43)	0.16	61 (45)	32 (51)	0.44	87 (47.3)	6 (40)	0.59
Item 11- Cognitive disorders	125 (62.8)	39 (58)	86 (65)	0.34	78 (57)	47 (75)	0.02 †	112 (60.9)	13 (87)	0.05
Item 12- Undernutrition	22 (11.5)	10 (15)	12 (10)	0.25	17 (13)	5 (8)	0.31	19 (10.7)	3 (20)	0.28
Item 13- Polymorbidity	108 (54.3)	40 (60)	68 (52)	0.27	74 (54)	34 (54)	0.95	100 (54.3)	8 (53)	0.94
Item 14- History of vertebral fractures	8 (4.0)	3 (5)	5 (4)	0.82	5 (4)	3 (5)	0.72	8 (4.3)	0 (0)	0.41
Item 15- Living alone	82 (41.4)	28 (42)	54 (41)	0.94	55 (41)	27 (43)	0.78	76 (41.5)	6 (40)	0.91
Item 16- Use anti-osteoporotic drugs	12 (6.0)	1 (2)	11 (8)	0.06	4 (3)	8 (13)	0.007 †	8 (4.3)	4 (27)	<0.001 †
25-hydroxyvitamin D, nmol/L (mean ± SD)	40 ± 23	16 ± 5	52 ± 18	<0.001 †	27 ± 13	68 ± 11	<0.001 †	36 ± 20	84 ± 7	<0.001 †

Data presented as *n* (%) where applicable; SD: standard deviation; *: Based on *t*-test or Chi-square test, as appropriate; †: *p*-value significant (i.e., <0.05) indicated.

**Table 2:** Univariate logistic regression models examining the cross-sectional associations between patients’ clinical characteristics and hypovitaminosis D (*n* = 199).

	Hypovitaminosis D								
	Severe Vitamin D Deficiency 25(OH)D ≤ 25 nmol/L			Vitamin D Deficiency 25(OH)D ≤ 50 nmol/L			Vitamin D Insufficiency 25(OH)D ≤ 75 nmol/L		
	OR	95% CI	*p*-Value	OR	95% CI	*p*-Value	OR	95% CI	*p*-Value
Item 1- Female gender	0.86	0.48–1.55	0.61	0.54	0.29–0.99	0.05	0.26	0.06–0.85	0.04 *
Item 2- Age, years	1.05	1.01–1.09	0.02 *	1.02	0.98–1.06	0.41	1.02	0.96–1.10	0.51
Item 3- Number of drugs daily taken	1.12	1.03–1.22	0.007 *	1.05	0.97–1.15	0.23	0.99	0.86–1.16	0.93
Item 4- Body mass index, kg/m^2^	0.95	0.89–1.02	0.15	1.02	0.96–1.10	0.55	0.96	0.87–1.08	0.51
Item 5- Use walking aids	1.50	0.86–2.61	0.16	1.20	0.68–2.14	0.54	0.98	0.37–2.79	0.97
Item 6- Use psychoactive drugs	1.01	0.55–1.83	0.98	0.64	0.35–1.17	0.15	0.31	0.09–0.90	0.04 *
Item 7- Wearing glasses	0.67	0.37–1.22	0.19	0.52	0.28–0.96	0.04 *	0.78	0.25–2.24	0.64
Item 8- Sad mood	1.12	0.59–2.10	0.72	0.70	0.37–1.33	0.27	0.49	0.17–1.45	0.19
Item 9- Fear of falling	1.24	0.69–2.24	0.47	1.08	0.60–1.99	0.79	0.67	0.23–1.95	0.46
Item 10- History of falls	1.53	0.85–2.77	0.16	0.79	0.43–1.43	0.44	1.35	0.47–4.16	0.59
Item 11- Cognitive disorders	0.75	0.41–1.37	0.34	0.46	0.23–0.87	0.02 *	0.24	0.04–0.90	0.07
Item 12- Undernutrition	1.75	0.70–4.31	0.22	1.66	0.62–5.24	0.34	0.46	0.13–2.15	0.26
Item 13- Polymorbidity	1.39	0.77–2.55	0.27	1.02	0.56–1.85	0.95	1.04	0.35–3.02	0.94
Item 14- History of vertebral fractures	1.19	0.24–5.01	0.82	0.76	0.18–3.82	0.72	-	-	0.99
Item 15- Living alone	1.04	0.57–1.88	0.91	0.91	0.50–1.67	0.75	1.06	0.37–3.26	0.92
Item 16- Use anti-osteoporotic drugs	0.17	0.01–0.89	0.09	0.21	0.05–0.69	0.01 *	0.13	0.03–0.53	0.002 *

CI: confidence interval; 25(OH)D: 25-hydroxyvitamin D; OR: odds ratio; *: *p*-value significant (i.e., <0.05) indicated.

**Table 3:** Metrological properties of the VDSP tool for the identification of hypovitaminosis D according to the different definitions of hypovitaminosis D (*n* = 199).

Hypovitaminosis D	True Positive	False Positive	True Negative	False Negative	Sensitivity, %	Specificity, %	Positive Predictive Value	Negative Predictive Value
Vitamin D insufficiency ≤ 75 nmol/L	8	31	153	7	53.3	83.2	20.5	95.6
Vitamin D deficiency ≤ 50 nmol/L	105	31	35	28	79.0	53.3	77.2	55.6
Severe vitamin D deficiency ≤ 25 nmol/L	58	53	79	12	86.2	60.0	93.8	38.5
